# The Development of Explicit and Implicit Game-Based Digital Behavioral Markers for the Assessment of Social Anxiety

**DOI:** 10.3389/fpsyg.2021.760850

**Published:** 2021-12-15

**Authors:** Martin Johannes Dechant, Julian Frommel, Regan Lee Mandryk

**Affiliations:** Human-Computer-Interaction Laboratory, Department of Computer Science, University of Saskatchewan, Saskatoon, SK, Canada

**Keywords:** social anxiety, assessment, digital biomarkers, digital games, interpersonal distance, in-game movement, behavioral markers

## Abstract

Social relationships are essential for humans; neglecting our social needs can reduce wellbeing or even lead to the development of more severe issues such as depression or substance dependency. Although essential, some individuals face major challenges in forming and maintaining social relationships due to the experience of social anxiety. The burden of social anxiety can be reduced through accessible assessment that leads to treatment. However, socially anxious individuals who seek help face many barriers stemming from geography, fear, or disparities in access to systems of care. But recent research suggested digital behavioral markers as a way to deliver cheap and easily accessible digital assessment for social anxiety: As earlier work shows, players with social anxiety show similar behaviors in virtual worlds as in the physical world, including tending to walk farther around other avatars and standing farther away from other avatars. The characteristics of the movement behavior in-game can be harnessed for the development of digital behavioral markers for the assessment of social anxiety. In this paper, we investigate whether implicit as well as explicit digital behavioral markers, proposed by prior work, for social anxiety can be used for predicting the level of social anxiety. We show that both, explicit and implicit digital behavioral markers can be harnessed for the assessment. Our findings provide further insights about how game-based digital behavioral markers can be used for the assessment of social anxiety.

## Introduction

Social anxiety, one of the most common mental problems worldwide (Kessler et al., 2005), is characterized by the intense fear of being exposed to the judgment of others. This intense fear may push affected individuals to withdraw from social interaction or can lead to elevated stress when facing an unavoidable social encounter (National Collaborating Centre for Mental Health, 2013; Karasewich and Kuhlmeier, 2020). But this intense fear of evaluation in social contexts stands in strong contrast to the universal human need to be part of a social group and form and maintain social bonds with others (Deci and Ryan, 2000; Taormina and Gao, 2013). As prior research shows, social anxiety is a highly prevalent mental illness worldwide and especially affects children and adolescents (Rao et al., 2007; Knappe et al., 2015). Unfortunately, the characteristic social inhibition and withdrawal of affected individuals are often mistaken for shyness by others or perceived as a character flaw by the individual (Heiser et al., 2009; Poole et al., 2017). Additionally, social anxiety and the underlying fear of any evaluation may cause individuals to avoid consultation with mental health professionals about their concerns (Lecrubier et al., 2000). As a result, only a minority of socially anxious individuals receive treatment (Ruscio et al., 2008). Individuals seeking help have to face not only the challenges of the mental disorder and the inherent social fears themselves, but also have to overcome sociocultural barriers, such as stigmatization and discrimination of mental health problems, economic barriers (Vigo et al., 2016), such as lack of financial coverage for mental health treatments, and geographical barriers (e.g., limited access to mental health care in remote communities) (Olfson et al., 2000; Kumar and Phookun, 2016).

The heavy burden of social anxiety may be decreased through early and easily accessible assessment; prior work showed that early assessment of social anxiety in adolescenthood—which is a critical time for the development of social anxiety (Velting and Albano, 2001; Rao et al., 2007)—increases the efficacy of treatment and could prevent the development of the most harmful effects (Dams et al., 2017). Prior research suggests evidence-based assessment of social anxiety as a reliable way to identify affected individuals (Silverman and Ollendick, 2005). This approach combines information gathered from multiple perspectives to characterize the patient’s concerns, develop suitable treatment strategies, and monitor the patient’s response to the selected treatment approaches (Tulbure et al., 2012). Assessment combines the personal perspective of the patient and the analysis of the patient’s behavior when confronted with social situations (Gordon et al., 2014). Mental health professionals utilize standardized questionnaires and interviews, in which the expert identifies concerns and the potential severity of social anxiety. While this approach is very effective in most cases, the subjective answers may be biased by several problems related to self-report measurements such as social desirability bias (Van de Mortel, 2008) and practice effect (Calamia et al., 2012). Within the context of social anxiety, these biases can often occur due to the desire of the patients to “please” the expert or to fit into the surrounding cultural and social context (Furnham and Henderson, 1982). To compensate for these effects, experts analyze behavioral data either by recording the reaction of the patient while being exposed to social situations within clinical assessment or by interviewing people close to the patient (Antony and Rowa, 2005). However, these approaches of behavioral information gathering are time consuming, can become expensive, and further involve additional people beyond the patient and the expert, which can introduce biases and false assumptions leading to biased conclusions (Davis, 2009; Ollendick and Davis, 2012). However, any accurate assessment is prevented when socially anxious individuals avoid reaching out for support because they are ashamed of their illness or in fear of negative evaluation (Lecrubier et al., 2000).

But prior research suggests a promising tool to assist existing techniques in assessing social anxiety: digital biomarkers. Digital biomarkers are measurable responses gathered from digital devices and used to reliably predict the incidence of a dysfunction or disorder (Gielis et al., 2019; Mandryk and Birk, 2019). Digital biomarkers can be harnessed to predict the severity of a disease and increase the temporal and spatial resolution of recorded behavior during an assessment (Dorsey et al., 2017). Furthermore, digital biomarkers are more resistant against human bias and may be less stress-inducing for patients (Shehab and Abdulle, 2011). Through various sensors [e.g., gaze tracking (Dechant et al., 2017)], platforms [e.g., social media platforms (Pourmand et al., 2019)], and devices [e.g., smartphones and wearables (Huang et al., 2016; Jacobson et al., 2020)], researchers can access a rich source of previously unobtainable insights, recorded with minimal effort from the patient and the expert alike (Torous et al., 2017). One subcategory of these digital biomarkers are digital behavioral markers which focus on the overall behavior, such as movement patterns using GPS coordinates (Mohr et al., 2017). More recently, motivated to harness the popularity of digital games, researcher have started to develop ways to incorporate game-based digital behavioral markers into the context of mental-health assessment (Dechant M. et al., 2021).

In 2019, two-thirds of the online population enjoy digital games (Wijman, 2020), including players of all ages, genders, and ethnicities from around the world. While enjoying digital games, players produce a large volume of data that can be used to create digital behavioral markers for the assessment of mental health concerns. Mandryk and Birk (2019) describe two complementary ways for developing game-based biomarkers: by harnessing in-game behavior data, which was produced naturally through interactions with the digital game or by developing custom games that place a player in a relevant context and record their response or performance using known behavioral correlates of mental health. Within the context of social anxiety, prior research outlined several reasons why behaviors related to social anxiety may translate into the context of a game: First, interpersonal interaction biases of the physical world have also been observed in similar player-avatar interactions (Bian et al., 2015); second, cognition biases and the characteristic avoidance behavior of social anxiety have been shown to manifest in-game as players of massively multiplayer online role player games (MMORPG) affected by social anxiety tend to avoid social interactions and highly challenging activities (Dechant et al., 2020). Third, prior research shows typical avoidance behavior of social anxiety in virtual reality simulations (Lange and Pauli, 2019). Finally, the media equation theory suggests that people react to computers with social responses (Nass and Moon, 2000; Rehm et al., 2016), which likely includes how socially anxious individuals react to virtual characters.

In developing digital behavioral markers, a first step is to identify behaviors that are characteristic of the mental health concern; the next step is to determine if these behaviors can be measured reliably by a system.

### Characteristic Behaviors of Social Anxiety

At the core of social anxiety is an intense fear of potential exposure to negative or positive evaluation from others (National Collaborating Centre for Mental Health, 2013; Spence and Rapee, 2016). This fear leads to changes in an individual’s behavior, for example social withdrawal, but also in physical symptoms such as blushing, trembling, or sweating. Prior work suggests that social anxiety is one of the most common anxiety disorders with a population prevalence of 2.0% (Fehm et al., 2005, 2008). This mental burden is most accurately expressed along a severity continuum where someone can experience a high level of social anxiety but not reach the threshold for a clinical diagnosis (Spence and Rapee, 2016). Several personal as well as environmental risk factors, such as social environment, can cause the development of social anxiety and affect how individuals could be affected by this mental burden. For example, people may experience high social anxiety only in a certain context, such as presenting in front of an unfamiliar audience, whereas the same task is less distressing with a familiar audience. Prior research emphasizes that adolescence is a critical phase for the development of social anxiety, which can grow into a chronic and generally unremitting course through a lifespan if left untreated (Perugi et al., 1999; Kessler, 2003; Villarosa-Hurlocker et al., 2019).

Socially anxious individuals face greater difficulties forming and maintaining social relationships with others (Miers et al., 2013). As a result of this fear, individuals tend to have fewer close friends, but are also at greater risk of being rejected or ignored by their peers. Additionally, affected individuals are also at higher risk of being victimized by others due to their lack of social competence (Navarro et al., 2012). As a way to escape this vicious circle for a moment, some socially anxious individuals develop dangerous coping strategies, such as substance abuse (Buckner et al., 2008). Untreated social anxiety can also lead to other severe mental illnesses, such as depression (Van Ameringen et al., 1991; Perugi et al., 1999).

As prior research shows, social inhibition and private anguish are inherent to social anxiety. However, these symptoms are often misinterpreted as shyness or as a character flaw of the individual rather than a mental illness (Poole et al., 2017; Jacobson et al., 2020). Also patients affected by social anxiety may avoid the consultation about their psychological problems with their physicians due to elevated experience of shame (Lecrubier et al., 2000). As a result, the nuanced effects of social anxiety are frequently underreported and under recognized. According to previous estimations, only 35% of individuals with symptoms of social anxiety disorder receive proper treatment for their anxiety (Ruscio et al., 2008). Therefore, to increase the efficacy and success rate of treatment, reliable assessment of social anxiety plays an important role in preventing serious harm (Herbert et al., 2009).

### Leveraging (Digital) Behavioral Markers for the Assessment of Social Anxiety

Mental health experts use standardized procedures that combine interviews, questionnaires, and behavioral information to achieve reliable assessment of mental health concerns. Most of the used scales, such as the Liebowitz Social Anxiety Scale (Liebowitz, 1987; Baker et al., 2002), depict different social scenarios and ask respondents to rate their fear and/or avoidance within the described situations. Other tools focus on the special needs of specific groups, such as children (Delgado et al., 2019). But there is an ongoing discussion about the advantages of evidence-based assessment, which harnesses not only self-reported perspectives but also relies on objective evidence for the mental illness. This search for evidence-based assessment approaches has fueled an interest in finding behavioral correlates that are also predictive of mental health. These behavioral correlates are often referred to as biomarkers, which are defined by the World Health Organization (WHO) as *“any substance, structure, or process that can be measured in the body or its products, and that influences or predicts the incidence of outcome or disease”* (Strimbu and Tavel, 2010). One example within the context of social anxiety would be the measurement of the individual’s blood pressure while being exposed to a social interaction. However, prior research provides only mixed results about the benefits of biomarkers for the assessment of social anxiety. *Digital Biomarkers* focus on objective, quantifiable physiological and behavioral data, which are collected and measured by means of various digital devices (e.g., wearable devices) (Jacobson et al., 2020).

To enhance the research agenda of finding reliable biomarkers for social anxiety, researchers have identified potential cognitive and behavioral characteristics of social anxiety which could be used for the assessment of social anxiety in a gaming task. Cognitive characteristics of social anxiety include aspects such as attentional biases (Pineles and Mineka, 2005) and interpretation (Miers et al., 2011) or memory biases (Glazier and Alden, 2019) that are affected by social anxiety. Prior work shows that socially anxious individuals prefer to maintain a greater distance from strangers (Givon-Benjio and Okon-Singer, 2020). Depending on the personality of an individual, these biases may cause the development of behavioral characteristics, such as the reliance and usage of avoidance and safety behaviors in the physical world (Voncken et al., 2006; McManus et al., 2008). These behaviors include individual approaches that aim to reduce the risk of drawing attention to oneself and therefore the risk of subsequent evaluation by others. For example, individuals with social anxiety over-rehearse a speech to prevent stuttering or other feared situations in front of an audience. But excessive reliance on safety behaviors results in consequences, such as the increased experience of anxiety, as well as self-focus attention, maintenance of negative beliefs, and even contamination of social situations, as the affected individual may come across as uninterested and insecure to others (Kley et al., 2012). Through various sensors, such as global positioning system (GPS) devices (Huang et al., 2016), gaze tracking devices (Chen et al., 2015), and skin conductance sensors (Voncken and Bögels, 2009), researchers successfully identified several promising digital biomarkers for social anxiety. Especially within virtual reality simulations, researchers have demonstrated the robustness of describing cognitive and behavioral markers for social anxiety, mostly by simulating physical situations in the safe space of virtual reality applications (Safir et al., 2012; Chesham et al., 2018). However, instrumenting people with contact sensors (e.g., gaze or skin conductance) limits the accessibility of the developed biomarkers. To gain the widest reach, digital biomarkers and behavioral markers should be extracted from standard interactions with computers or smartphones. One common source of engagement on computers or smartphones is through digital gaming.

Prior research has shown preliminary evidence that in-game behavior might be harnessed for the assessment of social anxiety (Dechant M. et al., 2021). In this earlier study, participants were asked to bypass a non-playable character (NPC) and move toward a targeted position in the room. Speed, accuracy, and path characteristics were measured per trial. Furthermore, two common game design aspects were altered to investigate design choices that may affect the visibility of typical social anxiety behaviors in the game. The results suggested that a third-person camera perspective combined with a customized self-representation resulted in the strongest effects of social anxiety on in-game behavior. People with higher levels of social anxiety tended to walk farther around the NPC and were less accurate in finding the final destination. This first investigation lays the essential foundation for further research about how digital behavioral markers in-game can be harnessed within digital games for the assessment of social anxiety. However, this initial work did not fully demonstrate the validity of in-game behaviors as a digital behavioral markers, as effects were shown on a trial level. To substantiate the initial evidence, we require further insights showing relationships per participant—a necessary approach for classifying individuals and suggesting the incidence of the mental health concern.

### The Present Research

Given the need for digital behavioral markers to assist with the assessment of social anxiety, the accessibility and prevalence of digital gaming, and preliminary evidence that behaviors characteristic of social anxiety also manifest in digital contexts, we conducted a study with the aim of identifying promising digital behavioral markers of social anxiety, drawn from interactions with a digital game.

Our game environment involves a player using a customized self-representation to interact with a NPC through a dialog, and to move around the NPC in the digital world. Based on previous work (Dechant M. et al., 2021), we look for the manifestation of movement behaviors, such as moving farther around an NPC. We also introduce new measures that rely on explicit behaviors (e.g., ask participants to indicate a comfortable distance to a stranger), and not just implicit ones (e.g., the movement behavior around a stranger), similar to prior work (Lange et al., 2008; Ritter et al., 2013). Explicit measurements are valuable for the prediction of deliberate and controlled behavior, whereas implicit measurements are assumed to be more indicative of less controlled and more impulsive behavior. Within the context of in-game digital biomarkers and behavioral markers for assessing social anxiety, prior work leveraged implicit measurements, which means that these behaviors are characteristic of less controlled, more impulsive, and rather autonomic in nature. However, an explicit measurement of behavior could also hold valuable insights for the development of further digital behavioral markers for the assessment of social anxiety. For example, *interpersonal distance (IPD)* describes the distance between an individual and a stranger at which individuals are comfortable to interact (Perry et al., 2015). Prior research shows that socially anxious individuals prefer to stand farther away in social interactions (Kroczek et al., 2020), resulting in a higher IPD. Therefore, the goal of this work is to answer the question of whether both explicit and implicit measurements of in-game behaviors can be harnessed as digital behavioral markers for the assessment of social anxiety. To be a reliable digital behavioral markers, measurements also must be able to identify affected individuals, a more challenging task than identifying characteristic behavior in a single trial. As such, we model the relationship between behavior and social anxiety at the level of participant.

## Materials and Methods

We conducted an experiment to investigate whether aspects of an in-game movement path as well as the interpersonal distance between player and an in-game character can be harnessed for the assessment of social anxiety of players. In the task, participants were asked to deliver documents to different teams in a building.

### Task Description

The task itself consists of two elements: The character editor and the assessment task; these elements will be described in the following sections.

#### Character Editor

Prior to the in-game task, participants were asked to create an avatar for the game, as prior work shows that customized avatars not only increase the engagement with a game but also affect the expression of social anxiety in-game (Dechant M. et al., 2021). First, participants selected the gender of their avatar (woman, man), then adjusted major aspects of the avatar’s body (i.e., height, weight, muscles, head offset, and breast size) and selected the hairstyle and skin and eye color of their avatar. Due to technical limitations of the underlying framework for the character creation, we were not able to offer a non-binary gender option in this experiment. Next, participants shaped the head of their avatar *via* 34 sliders to define different aspects of the head, such as face, eye, brows, nose, mouth, chin, jaw, ear, neck. After that, participants could choose the outfit of their avatar by selecting the style and color of clothing on the upper body, lower body, and shoes, as well as through head accessories, such as glasses, headphones, face masks, or hats (13 different options per element). To further enhance the participant’s identification with their avatar, we asked participants to describe the personality of their avatar by adjusting five sliders, which each represented one personality trait based on the Big Five Inventory. The 10 items of the BFI-10 (Rammstedt and John, 2007) were grouped into bipolar semantically anchored categories for the five traits and participants were asked to choose the slider position between the elements (e.g., calm to anxious). Participants had to spend at least 4 min with the design of their avatar. After 4 min, a button appeared on the screen that allowed participants to move to the next step of the experiment.

**FIGURE 1:**
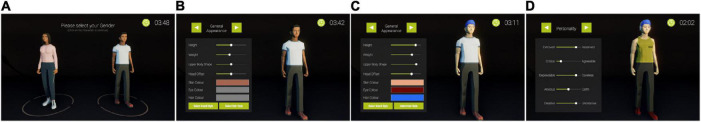
The character editor interface: First, participants selected the gender **(A)**, then customized their avatar’s body **(B)** and outfit **(C)**, and then selected their personality traits **(D)**. Images of the character assets reproduced with permission from Alex Lenk.

After customizing their in-game representation, participants filled out the Player Identification Scale (PIS) (Van Looy et al., 2012). Through the PIS, we were able to determine how much participants associated their avatar with themselves on the following three dimensions: similarity, embodied, and wishful identification. These insights were used to explain potential variance when participants may have felt they were limited in their self-expression due to the limitations of the character editor, such as the missing non-binary options as well as the lack of certain hair styles, such as curly hair styles. Figure 1 shows the character editor.

#### Assessment Task

**FIGURE 2:**
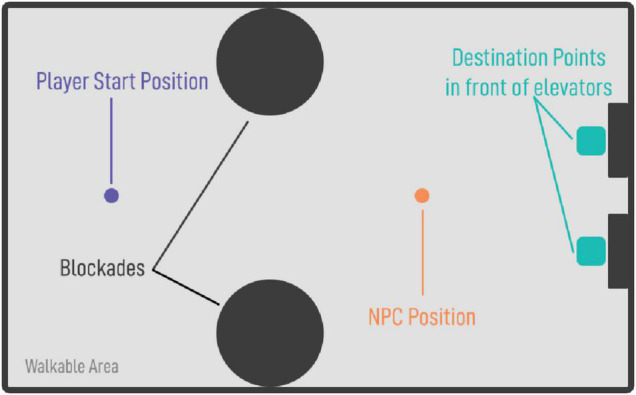
The setup of the of the assessment room: The avatar of the player stands at every trial on the purple point, the NPC on the orange point. Blockades (black) were used to guide the users’ movement toward the NPC.

In the beginning, participants were introduced to the background story and the control scheme of the task: As new interns at a large company, participants were instructed to deliver documents to 20 teams in a large building, named after Greek letters (e.g., Team alpha). Each team was randomly assigned to one floor number. These floors were then assigned to two elevators (1–10: right elevator; 11–20: left elevator). To find out the floor number, participants were instructed to walk toward a NPC, which stood ten meters in front of both elevators and centered in the room (see Figure 2), and to ask them on which floor they could find the team they were looking for. This interaction was intended to replicate a social situation with another stranger similar to prior work in the context of social anxiety assessments (Dechant et al., 2017).

**FIGURE 3:**
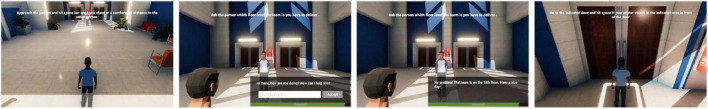
The task: First, participants have to approach the NPC and then ask for directions. Afterward they have to select the correct elevator. Images of the character assets reproduced with permission from Alex Lenk.

Once participants stood at a comfortable distance to the NPC, they had to press spacebar to start a conversation with the NPC. The NPC reacted either in a friendly manner, greeting participants and offering to help, or angry manner, insulting participants at the beginning of the conversation. In the conversation, the camera focused the face of the NPC and a chat interface appeared (see Figure 3). After either a friendly (e.g., “Hi! Nice to meet you. Let me know if I can help you.”) or angry (e.g., “What do you want, idiot?”) introduction, participants were asked to enter their question for the NPC, including the team name. Next, the NPC stated the floor number of the desired team in a friendly (e.g., “Team alpha is on floor 12”) or insulting manner (e.g., “Team alpha is on floor 12, idiot”). The two emotional reactions were balanced across the elevator sides over the experiment (five time for each side for each emotion). After the conversation, participants were asked to go to the elevator that led to the floor of the team. Once in front of the elevator, participants had to hit the space bar to confirm their selection. After that, we asked participants to rate the friendliness of the NPC on a scale from 0 (= “very angry”) to 100 (= “very friendly”). In total, participants had to deliver 20 files (2 elevator sides × 2 emotions × 5 repetitions). For each trial (one delivery of a file), we used a distinct team name. Prior work suggested the usage of a third-person camera setup, so that the participant can see over the shoulder of their own avatar over the whole trial to maximize in-game expression of social anxiety (because the self-representation is always visible on screen) (Bögels et al., 2002; George and Stopa, 2008). Whenever the player interacted with the NPC, the camera focused the NPC. Figure 3 summarizes the task implementation.

The task was implemented using the Unity Engine (Unity Technologies, 2020) combined with the Asset-Bundle “Advanced People Pack 2” (Lenk, 2020) and deployed in a web browser using the Bride of Frankenstein framework (Johanson, 2020). The asset package offers prepared 3D objects with blend shapes, which allowed us to adjust the body shape (e.g., weight and muscularity) as well as the face characteristics (e.g., the shape of the eyes). Furthermore, the package includes a suitable variation of clothing and hair styles for both genders that we could use for customization.

### Participants and Procedure

The experiment was deployed online, and participants were recruited using Amazon’s Mechanical Turk (MTurk). MTurk is an online platform, on which human intelligence tasks (HIT) can be offered by requesters. Workers can opt-in to these HITs. Prior researchers in different areas (Depping et al., 2018; Johanson et al., 2019; Passmore et al., 2020; Miller et al., 2021) successfully used this platform to collect data online. However, researchers need to carefully examine the data and ensure that bots or negligent workers are removed from the final analysis (Mason and Suri, 2012). Participants who were not able to play a 3D interaction game on their computers and those who left the experiment prior to completion were excluded from our data file. To avoid any behavioral bias induced by knowing the original goal of the story [e.g., white coat hypertension (Shehab and Abdulle, 2011)], we used a cover story to explain why participants were engaging in this task. For this experiment, we used the cover story that the goal of the experiment is to analyze effects of lag (input lag or network lag) on performance in a 3D game. The experiment itself as well as the usage of the cover story was approved by the Ethics board of the University of Saskatchewan. The actual goal of the experiment was revealed in the debrief of the study, at which point participants could ask to have their data excluded from further analysis.

MTurk allows requesters to use predefined or custom filters, such as demographics, prior work experience or how many HITs the worker successfully finished. For this study, we recruited participants who indicated that they live either in Canada or the United States of America, had at least 500 successfully completed HITs, and had a high approval rate of previous HITs (>95%).

We recorded the data of 117 participants following the suggestions of prior work (Dechant M. et al., 2021). First, we identified negligent participants based on their time spent answering the trait social anxiety questionnaire (LSAS; described later) and a short summary of the experiment, which they were required to provide in an open text field after the experiment. Participants who spent less than 1.5 s per item in the LSAS questionnaires were removed from the sample as well as participants who provided a non-descriptive or inaccurate summary of the experiment (e.g., “This is a nice experiment”). Furthermore, we removed participants who did not complete all 20 trials. After removing suspected bots and negligent participants (*n* = 11), we conducted our analysis with the remaining 102 participants (39 women, 62 men, one non-binary), aged 20–65 (M = 37.5, SD = 9.893). All participants received $8 USD compensation for participating in this study, which took approximately 40 min to be completed.

After providing consent, participants were provided with the cover story and had to answer one questionnaire to assess trait social anxiety and multiple about aspects of their gaming behavior, as well as their personal experience with lag in digital games (see section “Measurements”). These gaming questionnaires were used to emphasize the cover story. Following this, the task started with the previously introduced character creation interface, in which participants were instructed to design an avatar that represented them as best as possible. After that, the assessment task started, and participants completed their 20 trials. After the assessment task, demographic information was recorded, and participants were debriefed about the goal of the study. Additionally, we offered support resources such as contact information to a crisis hotline, and a link to picture of baby animals to reduce the potential negative effect on participants in considering their individual level of social anxiety.

### Measurements

#### Trait Social Anxiety

Participants answered the self-report version of the Liebowitz Social Anxiety Scale (LSAS) (Liebowitz, 1987; Baker et al., 2002). The LSAS consists of 24 items, split into two categories, which describe different social situations and interactions: social interaction (11 items; e.g., “Giving a party”) and public performance (13 items e.g., “Eating in a public place”). These items are rated on two 4-point Likert scales, one to measure the fear (0 = “none,” 3 = “severe”) and a second on how often these items were avoided in the last 2 weeks (0 = “0%/never,” 3 = “usually/68–100%”). The answers are summed together to estimate the level of trait social anxiety, between 0 and 144, where higher values indicate a higher level of social anxiety. Prior work suggests a score of 30 as a threshold to distinguish between non-anxious and anxious individuals, while a threshold of 60 provides the threshold that identifies a high risk of generalized social anxiety. In this sample, LSAS scores ranged from 1 to 126 (M = 58.7; SD = 28.65) with a high internal consistency (Cronbach’s α = 0.97). On average, our sample scored higher in comparison to a study with 31,243 cross-cultural participants (M = 44.07) (Caballo et al., 2019) as well as a study of 1,007 college students in the United Kingdom (M = 34.7) (Russell and Shaw, 2009); However, this study is in line with prior work examining United States MTurk samples [M = 51.24 (Dechant et al., 2020) and M = 58.85 (Dechant M. et al., 2021)], which contain workers with elevated levels of social anxiety.

#### Avatar Identification

In this experiment, we leveraged the avatar-related subscales of similarity, embodied identification, and wishful identification from the Player Identification Scale (PIS) (Van Looy et al., 2012). Participants rated their agreement to different statements such as “My avatar is like me in many ways” on 5-point Likert scales from 0 (= “strongly disagree”) to 4 (= “strongly agree”). Internal consistency was high for all subscales: similarity (Cronbach’s α = 0.93), embodied (Cronbach’s α = 0.94), and wishful identification (Cronbach’s α = 0.90).

#### Perceived Emotion of the NPC

After each trial, participants were asked to rate the perceived emotional state of the NPC on a scale ranging from 0 (= “very angry”) to 100 (= “very friendly”). This measurement was used to emphasize the reflection of the social interaction with the NPC.

#### In-Game Digital Behavioral Markers for Social Anxiety

To assess the level of social anxiety within the digital game, we recorded the following aspects of the participant’s in-game behavior: comfortable interpersonal distance, movement features, and temporal features.

##### Comfortable Interpersonal Distance

As previously described, participants were asked to approach the NPC and had to press the spacebar once they reached a comfortable distance to start a conversation. We calculated the Euclidian distance between the position of the participant’s avatar and the NPC when they hit spacebar to start the conversation with the NPC. This measurement is referred to as interpersonal distance (IPD). Prior research shows that individuals with social anxiety show a distance estimation bias. As a result of this bias, socially anxious individuals tend to prefer a larger IPD to other individuals, not only in the physical world, but also in virtual reality simulations (Lange and Pauli, 2019; Givon-Benjio and Okon-Singer, 2020; Kroczek et al., 2020). Therefore, we expected that elevated levels of social anxiety would result in a higher IPD.

##### Speed and Movement Features

We measured the time in seconds from starting the trial until participants completed the whole trial (*time spent in room*). This time measurement includes the time in the conversation and the rating screen in the end of the trial.

According to prior research, socially anxious individuals tend to walk farther around other individuals in the physical world as well as in simulated realities, such as virtual-reality experiences (Lange and Pauli, 2019). Furthermore, prior work suggests several aspects of the movement behavior in-game which can be used to identify social anxiety (Lange and Pauli, 2019; Dechant M. et al., 2021). Similar to prior work, we recorded the location of the participant’s avatar in the digital world with a timestamp, sampled every 50 ms after the conversation with the NPC was completed, as they approached the elevator.

Based on these samples, we calculated the following movement features, as suggested by prior work: The *path length* describes the absolute travelled distance per trial from the point where the conversation with the NPC started to one of the selected elevator doors.

**FIGURE 4:**
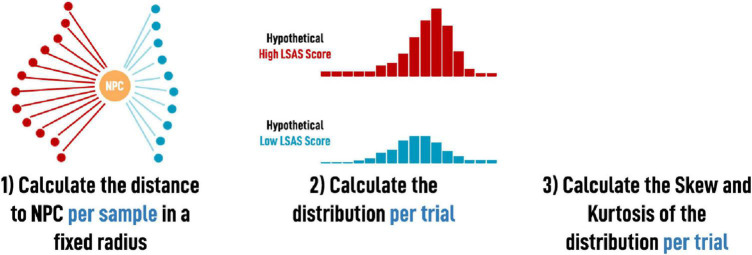
The calculation of skew and kurtosis of the distribution per trial.

Prior work shows that socially anxious individuals prefer to stay farther away from strangers in the physical realm (Givon-Benjio and Okon-Singer, 2020) and also in the digital realm (Kroczek et al., 2020). To confirm these findings, we measured the minimum distance between the participant’s avatar and the NPC after the conversation, measured as the smallest Euclidean distance to the NPC over the whole trial. We expected that participants with elevated social anxiety will show higher minimum distances as they prefer to stay farther away and walk farther around strangers (Lange and Pauli, 2019). The *mean distance to the NPC* represents the average distance to the NPC of all recorded samples per trial. Like prior work recommends, we additionally took all samples per trial and calculated two statistics related to the distribution of the samples: skew and kurtosis. Participants walking farther around the NPC should result in an elevated (right-leaning) skew. An elevated kurtosis is reflective of a narrower distribution, which results from a more consistent path with fewer points either very close to or very far from the NPC, indicative of less exploration and more controlled movements characteristic of people with elevated social anxiety (see Figure 4).

In summary, we had one explicit measurement, the (1) IPD, and six implicit measurements: (2) minimal and (3) mean distance to NPC, (4) time spent in room, (5) kurtosis and (6) skew of the distribution of the distances and the (7) path length.

#### Demographics

We recorded a variety of demographic factors, such as: age, gender, income, marital status, and ethnicity. See Table 1 for details about the characteristics of the sample.

**TABLE 1:** Overview of demographic information, LSAS and PIS measurements.

Variable	Categories	*N*	%	M	SD	Min	Max
Age		101		37.5	9.89	20	65
Gender	Woman	38	38.2				
	Man	62	60.8				
	Non-binary	1	1.0				
LSAS Score		102		58.7	28.65	1	126
Similarity Identification		102		3.015	0.7	0	4
Embodied Identification		102		2.65	0.87	0	4
Wishful Identification		102		2.27	0.95	0	4

### Hypotheses Based on Prior Work

Prior work suggests that highly socially anxious participants will aim to complete the task—which includes potential social threats and performance aspects—as quickly as possible, resulting in reduced time spent in the task (*time spent in room*). Further, elevated levels of social anxiety will result in elevated levels of *skew* and *kurtosis*, as participants with elevated social anxiety will try to walk farther around the NPC and have a higher *minimum* and *mean distance* to the NPC. As a result of this behavior, also the *path length* will increase as participants need to travel greater distances. Furthermore, we expect that the distance estimation bias caused by social anxiety (Givon-Benjio and Okon-Singer, 2020) will lead to an increased *IPD* for people with elevated levels of social anxiety.

### Analysis

Data were gathered and stored using an on-premises server and then exported once data collection was complete. All data were analyzed using SPSS 26; moderated regressions used the Process 3.4 integration. Statistical tests are described prior to reporting the results.

## Results

### Characteristics of the Sample

We first looked at the characteristics of the recorded sample and whether players feel represented and connected to their self-created in-game representation. Table 1 summarizes the characteristics of our sample of 102 participants. The PIS subscales revealed that players felt that the self-representation is similar to their own visual appearance, resulting in a high score of similar identification score compared to prior work (Dechant M. et al., 2021).

### RQ2: Is There an Influence of the NPC’s Emotion as Well as the Elevator Side?

First, we investigated whether the emotion of the NPC as well as the side of the destination elevator affected the explicit and implicit measurements. We used a repeated measures MANCOVA with side and emotion of NPC as repeated within-subject factors on the seven previously introduced dependent measures, controlling for age and gender. We found no significant results indicating that either the side of the elevator (all *p* > 0.129) nor the emotion of the NPC (all *p* > 0.148) significantly affected any movement behaviors. Therefore, we merged all trials together to calculate the central tendency, using the means of each measurement, and modeled the results on the participant level. Table 2 summarizes these results.

**TABLE 2:** Results of the MANCOVA.

Effect			Value	*F*	Hypothesis df	Error df	*p*	Partial Eta Squared
Between Subjects	Intercept	Pillai’s Trace	0.992	1,232.904	9	91	0.0	0.992
		Wilks’ Lambda	0.008	1,232.904	9	91	0.0	0.992
		Hoteling’s Trace	121.936	1,232.904	9	91	0.0	0.992
		Roy’s Largest Root	121.936	1,232.904	9	91	0.0	0.992
	Age	Pillai’s Trace	0.286	4.1	9	91	0.0	0.289
		Wilks’ Lambda	0.711	4.1	9	91	0.0	0.289
		Hoteling’s Trace	0.406	4.1	9	91	0.0	0.289
		Roy’s Largest Root	0.406	4.1	9	91	0.0	0.289
	Sex	Pillai’s Trace	0.182	2.252	9	91	0.025	0.182
		Wilks’ Lambda	0.818	2.252	9	91	0.025	0.182
		Hoteling’s Trace	0.223	2.252	9	91	0.025	0.182
		Roy’s Largest Root	0.223	2.252	9	91	0.025	0.182
Within Subjects	Emotion	Pillai’s Trace	0.067	0.726	9	91	0.684	0.067
		Wilks’ Lambda	0.933	0.726	9	91	0.684	0.067
		Hoteling’s Trace	0.072	0.726	9	91	0.684	0.067
		Roy’s Largest Root	0.072	0.726	9	91	0.684	0.067
	Side	Pillai’s Trace	0.3	0.316	9	91	0.968	0.030
		Wilks’ Lambda	0.970	0.361	9	91	0.968	0.030
		Hoteling’s Trace	0.31	0.361	9	91	0.968	0.030
		Roy’s Largest Root	0.31	0.361	9	91	0.968	0.030

### RQ3: Can We Predict the Participant’s Trait Social Anxiety Through in-Game Behavior?

To find out whether the proposed measures can be used for assessing the social anxiety of a player, we conducted hierarchical linear regressions for each measure to analyze whether it predicts trait social anxiety, expressed by the LSAS score (Liebowitz, 1987; Fresco et al., 2001). We included age and dummy-coded gender (−1 = man; 1 = woman, removed 1 non-binary participant) in the first block, and the movement feature in the second block, with LSAS as the dependent measure. We report unstandardized regression coefficients (denoted as B) and standardized regression coefficients (denoted as β). In all models for which the feature was significantly predicting social anxiety, the model fit was significant. Table 3 summarizes these results.

**TABLE 3:** The regression results.

	*B*	β	*p* (predictor)	*R*	*R* ^2^	*p* (model)
Interpersonal Distance (IPD)	**7.637**	**0.199**	**0.044**	**0.307**	**0.094**	**0.022**
Time Spent in Room	**0.386**	**0.229**	**0.023**	**0.308**	**0.095**	**0.01**
Kurtosis	**10.895**	**0.217**	**0.027**	**0.319**	**0.101**	**0.015**
Skew	**27.482**	**0.236**	**0.019**	**0.328**	**0.107**	**0.011**
Minimum Distance to NPC	**15.859**	**0.267**	**0.008**	**0.348**	**0.121**	**0.006**
Mean Distance to NPC	4.571	0.068	0.507	0.244	0.59	0.113
Path Length	1.299	0.093	0.379	0.249	0.062	0.1

*B denotes unstandardized regression coefficients.*

*β denotes standardized coefficients.*

*Significant results are bold.*

**FIGURE 5:**
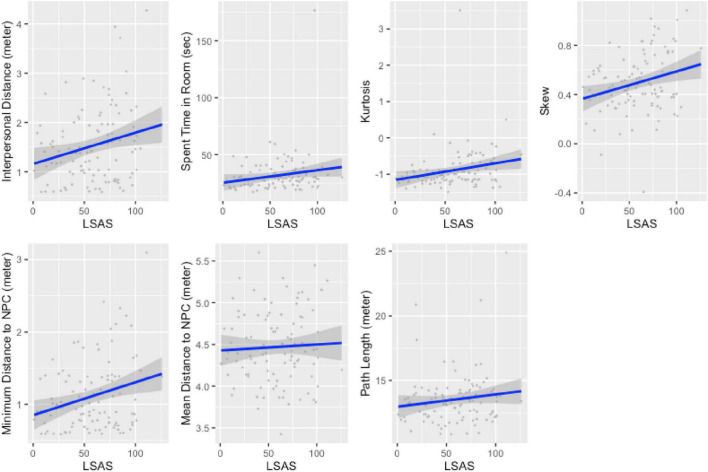
Scatter plots of the regression results for all proposed features.

The results show that most of the proposed in-game behavioral markers were useful for the prediction of trait social anxiety. As expected and suggested by the literature (Perry et al., 2015; Givon-Benjio and Okon-Singer, 2020), the interpersonal distance shows that players with elevated social anxiety explicitly prefer to stop at a larger distance when approaching an NPC. Furthermore, kurtosis and skew indicate that individuals with elevated social anxiety tend to maintain a consistently farther route around the NPC and avoid any close distance to the NPC, as the minimum distance to the NPC shows. Contrary to prior work (Dechant M. et al., 2021), the complete length of the path starting from the recording of the IPD to the selected elevator door, shows no significant relationship with LSAS. Also the mean distance between the player’s avatar and the NPC indicated no significant relationship with LSAS once modeled on the participant level. Figure 5 visualizes these results.

## Discussion

### Summary of Results

In summary, the results of this study confirm that characteristic behaviors of social anxiety appear within the realm of digital games. We show that higher levels of social anxiety result in a biased movement pattern expressed by an increased kurtosis and skew as well as increase in the minimum distance to the NPC. Additionally, we show that the characteristic preferences of social anxiety are also expressed by players prior to interacting with the NPC, as displayed in the increasing interpersonal distance. Contrary to prior work, the mean distance to the NPC as well as the path length around the NPC were not significant predictors for social anxiety.

### Explanation of Findings

In line with prior work, the results of this study show that explicit and implicit behaviors characteristic to social anxiety that have been observed in the physical world may also manifest in-game.

According to prior work in the physical world, as well as in virtual reality simulations, individuals with elevated social anxiety show that they prefer a larger physical distance between themselves and other individuals. Socially anxious individuals have also been shown to prefer less intimidating communication platforms, such as online chats (Miller et al., 2021) over voice or in person meetings. Within the context of digital games, players affected by social anxiety have been shown to be drawn toward games with a focus on playing together with others, such as MMORPGs; however, even in this context, the characteristic preference for distance toward others appears and biases the players’ preferences. Players with an elevated level of social anxiety withdraw from social activities in-game and avoid highly challenging tasks to avoid any potential exposure to the judgment of other players (Dechant et al., 2020). The results of our study contribute a similar result: socially anxious participants showed that they prefer greater distance between themselves and NPCs in-game, similar to within the physical world. However, this preference bias induced by social anxiety also appears in our study as what may be characterized as some visible strategies, similar to safety behavior techniques in the physical world. Prior work provides evidence for a distance estimation bias of socially anxious individuals. This bias may cause individuals to walk farther around another individual to not harm the personal space of another person as previously discussed. This study demonstrates similar behavior in the digital realm, which may stem from a similar strategy, or may be a habitual transfer of behaviors from the physical realm into the digital one.

A potential explanation for why social anxiety may trigger avoidance and preference biases that we observe in movement patterns may come from the elevated experience of self-awareness intensified by social presence; experiments in which socially anxious patients had to interact in front of a mirror show an association between experiencing social anxiety and elevated self-awareness (George and Stopa, 2008). This increased self-awareness plays an essential role in the maintenance of social anxiety, because socially anxious individuals are highly aware of their self-presentation to avoid any negative consequences (Bögels et al., 2002). In our study, the third-person camera perspective can be seen as a mirror which shows the player’s action within the digital realm of the game. Additionally, this effect is intensified through the use of a customized self-representation in the game. The combination of both resulted in prior work in the strongest expression of social anxiety within the game. Therefore, this study may provide more evidence for the hypothesis that customizing the own avatar and seeing it throughout the whole task enhances identification with the avatar, which triggers self-awareness and prompts the expression of safety behaviors.

Furthermore, the usage of customized avatars may enhance the experience of social presence (Bente et al., 2008). Social presence describes the experience that others are sharing the digital space with the player (Felnhofer et al., 2019). For the therapeutic effectiveness of digital interventions for social anxiety, the experience of social presence plays an essential role, because the experience of social presence may be more predictive of social anxiety responses than the self-reported experience of physical presence in a virtual world (Clutton-Brock et al., 2009). For example, prior work shows that participants experienced elevated social anxiety when their self-representation looked more similar to themselves (Vasalou et al., 2007; Aymerich-Franch et al., 2014). Also, social presence may also intensify the self-awareness of participants which may cause even stronger expression of social anxiety within the digital realm. Customizing avatars has also been recently shown to enhance fear within an exposure task for social anxiety (Dechant M.J. et al., 2021); the authors show that the customization enhances identification with the avatar, and speculate that customization enhances the invested effort and engagement in the task. In our study, all players customized their avatar, which may have helped reinforce the perception that they themselves were interacting with the NPC, aiding in the transference of behaviors and coping strategies common in physical spaces into our game.

Digital behavioral markers embedded in a gaming task may help to develop new approaches for the assessment of social anxiety, which may offer many benefits for health professionals as well as the affected individuals. Early assessment, such as when individuals are still young and developing their identities (Grant et al., 2005), habits, and behaviors, may prevent the harshest outcomes of social anxiety (Miers et al., 2013). However, a common challenge for the assessment is the risk of attrition due to waning of the patient’s motivation to continue with the process. Game based assessment may help to increase the engagement of patients who enjoy playing video games (Birk et al., 2016; Mandryk and Birk, 2019), which is not only limited to younger generations, as the still growing popularity of digital games in older generations suggests (ESA, 2019). These customized games may offer an easily accessible screening tool in combination with other existing tools as well as a way for continuous monitoring of the current progress in a treatment. However, researchers as well as developers need to be aware of several ethical implications when harnessing digital biomarkers in a gaming task.

### Ethical Implications

Despite the suggested benefits of digital biomarkers in general within a digital game, the growing discussion about the appropriate usage of data derived from digital sources raises several ethical concerns that are part of an ongoing discussion (Floridi and Taddeo, 2016; Metcalf and Crawford, 2016; Mittelstadt et al., 2016; Kenwright, 2018). When harnessing digital biomarkers, we need to ensure the protection of the user’s privacy as well as the creation of a safe space for the patient grounded on a legal basis. The communication about measured (mental) health issues might have negative effects for the patient, and experts need to be aware of the potential harm the communication can cause for the player. When deployed by mental health experts, assessment data is interpreted within a safe context that can lower the risk for a biased interpretation of the results of the assessment. Additionally, to leverage these approaches in an appropriate and healthy manner, developers must ensure the safety of the patient as well as the therapist while using such tools.

However, designers of these applications need to balance the enjoyment of the digital game and the requirements of an effective assessment. Depending on the complexity of the game mechanics as well as the context of the game, players may behave differently in the digital realm as compared to the physical. As prior work outlines, games allow players to explore and enact different roles or fantasies, which guides them to behave differently. Furthermore, excessive play of video games should be avoided especially within the context of social anxiety. Prior work emphasizes a relationship between problematic gaming behavior and social anxiety (Cole and Hooley, 2013; Lee and Leeson, 2015). However, this relationship highlights the potential of using game-based behavioral markers applied by medical experts for assessing social anxiety. Overall, these techniques should only be applied within a health and safe patient-therapist relationship.

### Limitations and Future Research

There are several questions future research may address in further follow-up investigations. First the lack of non-binary representation; although only one participant identified themself as non-binary, future research should overcome the technical limitations of the used framework to offer participants a non-binary character option, which will allow them to identify themselves with their character. Second, we must consider the influence of culture on the expression of social anxiety. This work focused on the development of digital b behavioral markers for characteristics linked to social anxiety in a western context. However, the literature suggests the differentiation of social anxiety and *Taijin kyofusho* (Kleinknecht et al., 1997) which, in comparison to social anxiety (disorder), focuses on the fear of embarrassing or *offending another person* rather than *embarrassing oneself* in front of others. Furthermore, the preference of the interpersonal distance may also be biased by the cultural background, where the shape of what constitutes personal space varies over different cultures. Therefore, future research may investigate the effect of the cultural background on the expression of social anxiety behavior within the digital realm. Third, we acknowledge the lack of comparison to other mental illnesses. Prior research suggests a comorbidity between different mental illnesses, such as Autism and Social anxiety. Therefore, future work may investigate if related mental illnesses will be expressed differently through the proposed in-game digital behavioral markers. However, this paper further confirms that prior suggested in-game movement behaviors and preference features embedded into a game can be used for assessing the personal level of social anxiety.

## Conclusion

Social anxiety, one of the most common mental disorders worldwide, is underdiagnosed due to several barriers induced by the disorder as well as by existing assessment approaches. However, prior work suggests digital behavioral markers embedded in a gaming task to complement existing assessment techniques. These behavioral markers allow for a timely identification for early intervention, ongoing assessment during an intervention and lowering barriers to access systems of care, due to their broad accessibility and low cost. This study replicated previous findings that movement behaviors characteristic of people with social anxiety also manifest in digital spaces, and also provides new evidence that digital behavioral markers within a game can be harnessed to assess the personal level of social anxiety. Specifically, the player’s preferred distance from an NPC prior to interaction as well as altered movement path around the NPC after interaction were able to predict social anxiety. However, certain aspects of the movement path, such as the length of the path, were less useful as they were strongly affected by the surrounding game environment. The presented findings confirm prior research showing how game-based digital behavioral markers can be effectively used to assess social anxiety and offering the benefits of early and ongoing digital assessment for the mental health expert and the patient.

## Data Availability Statement

The raw data supporting the conclusions of this article will be made available by the authors, without undue reservation.

## Ethics Statement

The studies involving human participants were reviewed and approved by Ethics Board of the University of Saskatchewan. The patients/participants provided their written informed consent to participate in this study.

## Author Contributions

MD led the research, co-designed the task and experiment, implemented the task, gathered and analyzed the data, and wrote the manuscript. JF provided feedback on the task and experiment design, contributed to the analyses, and edited the manuscript. RM co-designed the task and experiment, guided the analyses, and edited the manuscript. All authors contributed to the article and approved the submitted version.

## Conflict of Interest

The authors declare that the research was conducted in the absence of any commercial or financial relationships that could be construed as a potential conflict of interest.

## Publisher’s Note

All claims expressed in this article are solely those of the authors and do not necessarily represent those of their affiliated organizations, or those of the publisher, the editors and the reviewers. Any product that may be evaluated in this article, or claim that may be made by its manufacturer, is not guaranteed or endorsed by the publisher.
